# TMEM199-Congenital Disorder of Glycosylation With Novel Phenotype and Genotype in a Chinese Boy

**DOI:** 10.3389/fgene.2022.833495

**Published:** 2022-03-24

**Authors:** Yuan Fang, Kuerbanjiang Abuduxikuer, Yi-Zhen Wang, Shao-Mei Li, Lian Chen, Jian-She Wang

**Affiliations:** ^1^ Department of Pathology, Anhui Provincial Children’s Hospital, Hefei, China; ^2^ Department of Hepatology, Children’s Hospital of Fudan University, Shanghai, China; ^3^ Department of Pathology, Children’s Hospital of Fudan University, Shanghai, China

**Keywords:** congenital disorders of glycosylation, TMEM199-CDG, inherited metabolic disease, liver disease, mutation

## Abstract

**Background:** TMEM199-congenital disorder of glycosylation (TMEM199-CDG) is a rare autosomal recessive inherited disease characterized by chronically elevated serum transaminase, decreased serum ceruloplasmin, steatosis and/or fibrosis, *TMEM199* mutation, reduced level of TMEM199 protein, and abnormal protein glycosylation.

**Methods:** The information of a Chinese patient with TMEM199-CDG in the Children’s Hospital of Fudan University was reviewed. The patient’s clinical, pathological, and molecular features were obtained by clinical data study, liver biopsy, immunohistochemistry, and molecular genetic analysis.

**Results:** A 4-year-old Chinese boy presented with hypertransaminasemia, hypercholesterolemia, elevated alkaline phosphatase, decreased serum ceruloplasmin and serum copper level, and coagulopathy since birth. To the best of our knowledge, novel findings included strabismus, cirrhosis by liver biopsy, reduced expression of TMEM199 by immunohistochemistry, and a frameshift variant of c.128delA/p.Lys43Argfs*25 in the *TMEM199* gene.

**Conclusion:** This case added to the phenotypic and genotypic spectrum of TMEM199-CDG.

## Introduction

Congenital disorder of glycosylation (CDG) is an emerging group of metabolic diseases with autosomal recessive inheritance. One or more glycosylation enzyme defects lead to the abnormal glycosylation of protein or lipid in CDG, resulting in corresponding clinical symptoms (Yarema and Bertozzi, 2001; Jaeken, 2013). Common manifestations include dysmorphic features; diarrhea or malnutrition; abnormalities in the liver, coagulation, gland, and immune and nervous system; psychomotor disability; language retardation; ataxia; eye abnormalities; spinal or joint impairment; and cardiac disorders. Given the diversity of clinical performance, definitive diagnosis of CDG is difficult. With the development of whole-exome sequencing (WES), more than 140 CDG types have been identified. As a new subtype discovered in recent years, TMEM199-CDG (OMIM: 616829) is characterized by isolated or predominant liver involvement, which is different from most CDGs associated with multiple organ dysfunction (Marques-da-Silva et al., 2017). To date, only seven cases of TMEM199-CDG have been reported worldwide (Jansen et al., 2016; Vajro et al., 2018). The patients presented with cryptogenic elevated alanine aminotransferase, aspartate aminotransferase, alkaline phosphatase, creatine kinase, total cholesterol, low-density lipoprotein cholesterol, and decreased serum ceruloplasmin. Liver biopsies revealed steatosis and/or fibrosis, and an ultrastructure of dilation and vesiculation of the Golgi and/or endoplasmic reticulum (ER). Several mutation sites of *TMEM199* were found by sequencing. Western blot confirmed reduced or undetectable levels of TMEM199 protein. Glycosylation analysis results were consistent with the type Ⅱ CDG pattern.

Herein, we reported a Chinese boy with TMEM199-CDG, presenting with abnormal liver function, strabismus, and mild psychomotor delay. The liver biopsy showed steatosis, severe fibrosis as cirrhosis at stage 4, and reduced expression of TMEM199 by immunohistochemistry. WES revealed two compound heterozygous variants, c.20C > A mutation and c.128delA deletion in *TMEM199* exon 1, of which the latter had not been previously reported. Treatments with bicyclic alcohol, zinc sulfate, and penicillamine yielded no effect, while no significant progression in the course of the disease was observed after 1-year follow-up. To the best of our knowledge, he is the first Chinese patient described with TMEM199-CDG who carried a novel phenotype and genotype, which might expand the understanding of this rare disease.

## Materials and Methods

### Patient

The information of a Chinese pediatric patient with TMEM199-CDG in the Children’s Hospital of Fudan University was reviewed. Clinical, pathological, and molecular features of the patient were assessed by clinical manifestations, laboratory investigations, liver biopsy, immunohistochemistry, and molecular genetic analysis. This study conformed to the provisions of the institutional ethics committee and the Declaration of Helsinki (as revised in 2013). The patient’s parents shared all procedures including treatment and signed the written informed consent. The written informed consent was obtained for publication of any potentially identifiable images or data included in this article.

### Liver Biopsy

The specimen was obtained from core-needle biopsy, fixed in 10% buffered formalin, dehydrated in graded concentrations of ethyl alcohol, and embedded in paraffin. Then it underwent routine staining for hematoxylin and eosin (HE), and histochemical special staining for periodic acid–Schiff (PAS) with and without diastase, Masson, reticulin, copper, and iron.

### Immunohistochemistry

Additional 4-µm sections were deparaffinized, rehydrated, and pretreated with 3% H_2_O_2_ to eliminate endogenous peroxidase activity. Moreover, they were treated with EDTA (pH 9) or citrate buffer (pH 6) for heat-mediated antigen retrieval before commencing with the immunohistochemical staining protocol. The primary antibodies used included CK7 (clone MX053, ready-to-use solution), CK19 (clone MX054, ready-to-use solution), CD68 (clone MX075, ready-to-use solution), CD163 (clone MX081, ready-to-use solution), CD3 (clone MX036, ready-to-use solution), and CD8 (clone C8/144B, ready-to-use solution), which were purchased from http://www.maxim.com.cn (Fuzhou, China). In addition to the series of conventional antibodies, a rabbit polyclonal antibody against TMEM199 (purchased from http://www.abcam.cn, product code: ab121907) with a 1:50 dilution was utilized. The sections were incubated overnight at 4°C, followed by incubating with a general secondary antibody for 1 h at room temperature. Finally, the sections were developed with DAB and counterstained with hematoxylin. A normal liver sample (donated by a surgical patient) was prepared as the positive control, while omitting the first antibody as the negative control.

### Molecular Genetic Analysis

Genetic testing was performed by Running Gene Inc (http://www.mono-mz.com/auth, Beijing, China). EDTA-anticoagulated whole blood specimens were collected from the patient and his parents. DNA was isolated from peripheral blood using the DNA Isolation Kit (Blood DNA Kit V2, CW2553). Concentrations were determined on a Qubit fluorometer (Invitrogen, Q33216) using the Qubit dsDNA HS Assay Kit (Invitrogen, Q32851). Agarose gel (1%) electrophoresis was performed for the quality control. A total of 1 μg isolated DNA was sheared using the following parameters: duty cycle 10%, intensity 5, cycles per burst 200, and time six cycles per 60 s. This step was performed using a Bioruptor UCD-200 (Diagenode). All samples were sheared very consistently, and the size distribution peak was around 200 bp. In total, 3 μl of the sheared DNA was electrophoresed in a 2% agarose gel to confirm the presence of fragments of the desired size range. The multiplex ligation-dependent probe amplification assay (MLPA) was performed using the *ATP7B* MLPA kit (SALSA MLPA Probemix P098-D1, MRC-Holland, the Netherlands) (Chen et al., 2019). However, MLPA analysis excluded copy number variations in *ATP7B*. Then IDT-WES was performed to dig for potential genetic abnormalities. DNA libraries were prepared using the KAPA Library Preparation Kit (Kapa Biosystems, KR0453) following the manufacturer’s instructions. The procedure comprised three standard steps: end-repair of fragmented DNA, A-tailing, adapter ligation, and amplification. Purifications between steps were carried out using Agencourt AMPure XP beads. The libraries were estimated using the Qubit dsDNA HS Assay kit (Invitrogen, Q32851). Hybridization of pooled libraries to the capture probes and removal of non-hybridized library molecules were carried out according to the SeqCap hybrid Mix system. DNA libraries were sequenced using the Illumina novaseq platform (Illumina, SanDiego, United States) as paired-end 200-bp reads. For data analysis, adapters and low-quality sequences were removed to obtain clean and basic data. Genome Analysis Toolkit (GATK) (https://gatk.broadinstitute.org) was used for variation calling to summarize single-nucleotide variants (SNVs) and indels. ANNOVAR software and Enliven® Variants Annotation Interpretation System were employed for annotation and interpretation. Data were aligned against the Human Gene Mutation Database (HGMD) Professional (http://www.hgmd.cf.ac.uk), 1,000 Genome Database (www.1000 genomes.org), Genome Aggregation Database (gnomAD) (https://gnomad.broadinstitute.org), dbSNP152 (https://www.ncbi.nlm.nih.gov/snp), and Exome Aggregation Consortium (ExAC) (http://exac.broadinstitute.org). The variants were filtered out only with a population frequency of <5% in 1,000 genomes and <2% in gnomAD. Damage prediction of the genetic variants was conducted by Combined Annotation-Dependent Depletion (CADD) (https://cadd.gs.washington.edu) for scoring and mutation significance cutoff (MSC) (https://lab.rockefeller.edu/casanova/MSC) for further comparison. The MSC server was applied to CADD, PolyPhen 2 (http://genetics.bwh.harvard.edu/pph2) and SIFT (https://sift.bii.a-star.edu.sg/www/SIFT_indels2.html), with a confidence interval of 99% and database source of HGMD and ClinVar (https://www.ncbi.nlm.nih.gov/clinvar). Genomics England PanelApp (https://panelapp.genomicsengland.co.uk), a crowdsourcing tool, was utilized for analysis based on variant–disease and gene–disease associations. Human Phenotype Ontology (HPO) (https://hpo.jax.org), Online Mendelian Inheritance in Man (OMIM) (https://www.omim.org), and HGMD database were used to match the phenotype descriptions with variant and gene prioritization results. According to the American College of Medical Genetics and Genomics (ACMG) guidelines, genetic variants were classified as pathogenic, likely pathogenic, variants of uncertain significance (VUS), likely benign, and benign. Thereafter, two pathogenic variants of *TMEM199* were identified with the transcript of NM_152464.1, which was validated by using Sanger sequencing. The pathogenicity of amino acid changes caused by gene mutations was predicted using MutationTaster (http://www.mutationtaster.org). We conducted protein modeling using the SWISS-model (https://www.swissmodel.expasy.org) with a UniProtKB code Q8N511, and the mutated structures were analyzed and visualized using PyMol (http://www.pymol.org).

## Results

### Clinical History

The patient was born to healthy non-consanguineous parents as the second child of the family, whose older sister was healthy. At the age of 1 month, the patient was hospitalized for jaundice. During a routine blood test, remarkably elevated aspartate aminotransferase (253.0 IU/L, reference range 0–40 IU/L), γ-glutamyl transferase (540.0 IU/L, reference range 7–50 IU/L) and total bilirubin (303.8 μmol/L, reference range 5.1–17.1 μmol/L), and decreased globulin (9.6 g/L, reference range 20–30 g/L) and ceruloplasmin (0.04 g/L, reference range 0.22–0.58 g/L) were noticed. The patient was discharged after jaundice was treated. However, the hypertransaminasemia, hypercholesterolemia, elevated alkaline phosphatase, decreased serum ceruloplasmin and serum copper level, and coagulopathy persisted during the approximate 4-year follow-up.

Then the 4-year-old boy with abnormal liver function since birth was admitted to our in-patient department. Physical examination showed that his height was 108 cm (87th percentile by the WHO standard) and weight was 19 kg (88th percentile). Ocular strabismus was present, while the Kayser–Fleischer ring was absent. The liver was located 3 cm below the right costal and 4 cm below the xiphoid process, with a soft texture. The examination of the skin, abdomen, and nervous system showed no abnormalities. Abdominal ultrasonography indicated that the echogenicity of the liver was inhomogeneous, showing mild fatty liver manifestation. Cranial magnetic resonance imaging revealed that the anterior part of the right ventricle was slightly plentiful, and part of the sinus mucosa was thickened. The patient was assessed as having a mild intellectual disability by the development screening test (DST). The measured intellectual age was below his actual 51 months, showing 36 months in exercise, 27 months in social adaptation, and 36 months in intelligence. The majority of liver enzymes were elevated, including alanine aminotransferase (58.80 IU/L, reference range 0–40 IU/L), aspartate aminotransferase (123.20 IU/L, reference range 0–40 IU/L), alkaline phosphatase (1296.00 IU/L, reference range 42–383 IU/L), and creatine kinase isoenzyme (30.8 IU/L, reference range 0–30 IU/L). The lipid profile revealed increased total cholesterol (7.92 mmol/L, reference range 3.1–5.2 mmol/L), high-density lipoprotein cholesterol (1.61 mmol/L, reference range 0.91–2.05 mmol/L), and low-density lipoprotein cholesterol (5.72 mmol/L, reference range 1.30–3.90 mmol/L). Serum ceruloplasmin (0.04 g/L, reference range 0.22–0.58 g/L) and serum copper (2.80umol/L, reference range 10.99–21.98 μmol/L) levels were decreased. Transferrin (2.7 g/L, reference range 2.5–4.3 g/L) and ferritin (48.02 ng/ml, reference range 30–400 ng/ml) levels were normal. With the exception of prolonged activated partial thromboplastin time (46.6 s, reference range 28.0–44.5 s), increased D-dimer (0.57 mg/L, reference range 0–0.3 mg/L) and decreased fibrinogen (1.43 g/L, reference range 2–4 g/L), other coagulation indicators were normal. Table 1 summarizes the predominant laboratory investigations of the patient in different phases. Subsequently, the patient was treated with bicyclic alcohol, zinc sulfate, and penicillamine for 1 year. To date, there has been no significant improvement or disease progression during the surveillance period. The follow-up showed elevated serum transaminases, alkaline phosphatase, and lipid profiles, and persistent low ceruloplasmin.

**TABLE 1:** Predominant changes in laboratory investigations of the Chinese patient.

Age (m, month)		1 m	1.6 m	1.8 m	2.5 m	5.6 m	8.7 m	49.3 m	50.9 m	56.1 m	57.1 m	59.6 m	63.3 m
Serum biochemistry (reference range)	Albumin (35–55 g/L)	45.0	45.0	54.0	44.0	48.0	47.0	50.0	48.2	49.1	46.2	50.3	48.6
	Globulin (20–30 g/L)	9.6	15.0	18.0	14.0	14.0	16.0	NA	NA	14.6	18.3	12.8	14.1
	Alanine aminotransferase (0–40 IU/L)	63.0	88.0	69.0	80.0	99.0	129.0	156.5	58.8	297.7	169.4	77.7	128.6
	Aspartate aminotransferase (0–40 IU/L)	253.0	247.0	186.0	207.0	257.0	746.0	150.0	123.2	196.3	108.3	62.9	114.0
	Total bilirubin (5.1–17.1 μmol/L)	303.8	147.3	108.0	117.5	19.2	10.3	7.3	4.3	4.6	3.5	2.0	5.0
	Direct bilirubin (0–6 μmol/L)	14.3	14.9	19.7	14.5	8.5	6.2	0.9	NA	1.7	2.1	1.0	1.5
	γ-glutamyl transferase (7–50 IU/L)	540.0	557.0	459.0	497.0	201.0	343.0	22.6	25.7	25.0	19.0	18.0	17.0
	Total bile acid (0–10 μmol/L)	24.9	30.0	43.0	54.0	124.0	200.0	NA	2.8	3.1	12.6	7.7	8.7
	Alkaline phosphatase (42–383 IU/L)	410.0	757.0	974.0	700.0	769.0	789.0	1030.2	1296.0	1035.0	692.0	918.0	946.0
	Blood glucose (3.9–5.8 mmol/L)	NA	3.3	3.4	3.8	2.9	2.8	4.6	4.9	4.87	NA	NA	4.67
	Lactic acid (0–2 mmol/L)	NA	1.41	NA	NA	NA	NA	NA	NA	NA	NA	NA	NA
	Ammonia (10–47 μmol/L)	NA	24.8	NA	NA	NA	NA	NA	55.0	NA	NA	NA	NA
	Total cholesterol (3.1–5.2 mmol/L)	NA	7.74	9.30	7.00	6.73	8.73	NA	7.92	6.09	NA	NA	6.52
	LDL cholesterol (1.30–3.90 mmol/L)	NA	4.89	NA	NA	4.36	7.17	NA	5.72	4.66	NA	NA	4.93
	HDL cholesterol (0.91–2.05 mmol/L)	NA	3.03	NA	NA	2.46	1.17	NA	1.61	1.01	NA	NA	1.19
	Triglyceride (0.56–1.70 mmol/L)	NA	1.04	0.75	0.50	0.87	2.05	NA	0.90	0.82	NA	NA	0.66
	Procalcitonin (<0.05 ng/ml)	0.22	0.20	NA	NA	NA	0.54	NA	NA	NA	NA	NA	NA
	Ceruloplasmin (0.22–0.58 g/L)	0.04	0.03	0.04	NA	0.05	0.12	0.06	0.04	0.07	NA	NA	0.07
	Serum Copper (10.99–21.98 μmol/L)	NA	NA	NA	NA	4.0	8.6	2.8	2.8	NA	NA	NA	NA
Blood coagulation profiles (reference range)	Activated partial thromboplastin time (28.0–44.5 s)	53.8	39.2	NA	NA	NA	NA	NA	46.6	NA	NA	NA	34.5
	D-dimer (0–0.3 mg/L)	NA	NA	NA	NA	NA	NA	NA	0.57	NA	NA	NA	NA
	Fibrinogen (2–4 g/L)	1.76	1.67	NA	NA	NA	NA	NA	1.43	NA	NA	NA	1.64
	Thrombin time (14–21 s)	21.5	18.9	NA	NA	NA	NA	NA	17.0	NA	NA	NA	15.3
	International normalized ratio (0.8–1.2)	1.03	0.96	NA	NA	NA	NA	NA	1.09	NA	NA	NA	1.08
	Prothrombin time (12.0–14.8 s)	11.6	11.1	NA	NA	NA	NA	NA	14.4	NA	NA	NA	13.1
	Prothrombin time activity (80–100%)	85.4	83.1	NA	NA	NA	NA	NA	83.0	NA	NA	NA	88.0

### Liver Biopsy

**FIGURE 1:**
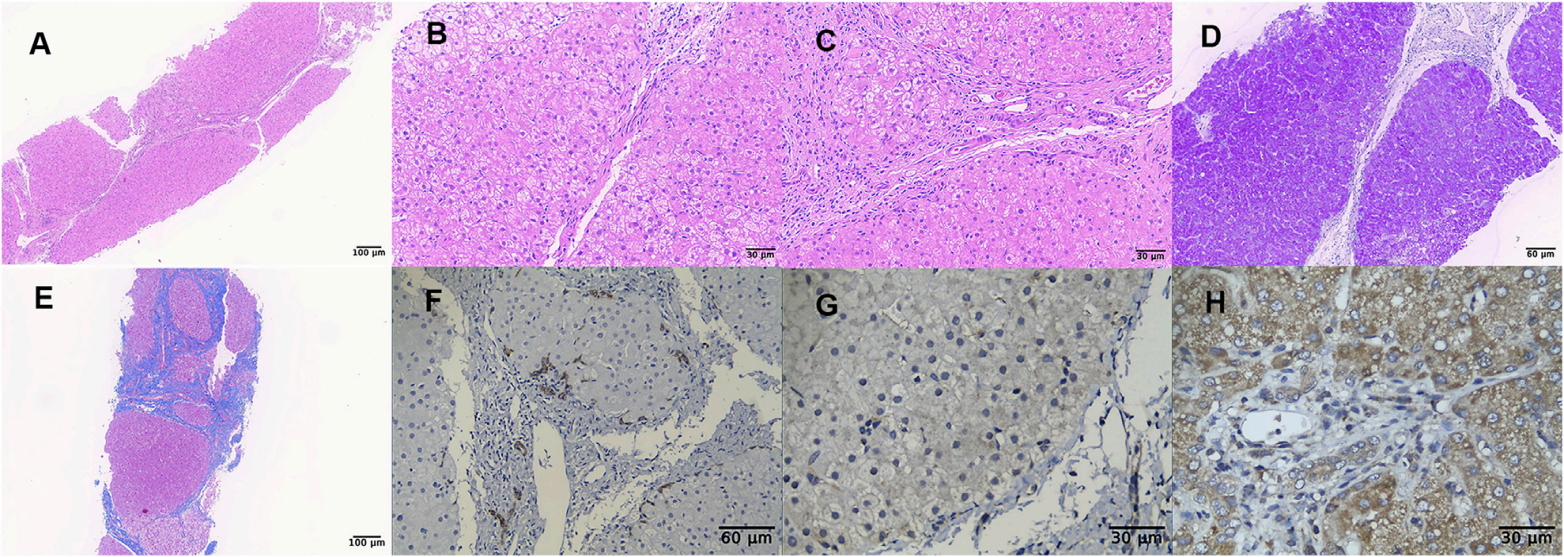
Liver biopsy (all originally magnified principal images). Hematoxylin and eosin staining showed the discorded architecture of liver lobules and the formation of pseudo-lobules **(A×100)**, ground-glass-like hepatic cells with lightly stained cytoplasm and obvious vesicular steatosis in portal area **(B×400)**, and lymphocytic infiltration in the portal area **(C×400)**. PAS staining identified small vacuoles in partial hepatocytes **(D×200)**. Formation of bridging-like fibrosis and pseudo-lobule was highlighted by Masson staining **(E×100)**. Mild hyperplasia of bile ducts along the margins of pseudo-lobule was showed by immunohistochemical staining of CK7 **(F×200)**, while weaker staining in the index patient **(G×400)** than in normal control **(H×400)** was observed by TMEM199 immunohistochemical detection.

Liver biopsy showed that the architecture of liver lobules was disordered, and pseudo-lobule was formed (Figure 1A). High power exhibited ground-glass-like hepatic cells, with a lightly stained cytoplasm and clear cell membrane, and vesicular steatosis, which were more evident in the portal area, rather than the regions around the central vein (Figure 1B). Infiltration of few lymphocytes in the portal area was also observed (Figure 1C). PAS staining identified small vacuoles in partial hepatocytes (Figure 1D), while PAS with diastase, copper, and iron staining were negative. The hyperplastic fibrous tissue in the portal area separated liver lobules, formatting bridging-like fibrosis and pseudo-lobules, which was highlighted by Masson staining (Figure 1E). The preserved reticular scaffold structure was displayed by reticulin staining. It presented cirrhosis with stage 4 assessment according to the Scheurer histopathologic scoring system (Scheuer, 1991).

### Immunohistochemistry

Immunohistochemical staining of CK7 and CK19 showed mild hyperplasia of bile ducts along the margins of pseudo-lobules (Figure 1F). Slightly proliferated Kupffer cells in hepatic sinusoids were indicated by CD68 and CD163 staining, and lymphocytes infiltrating in the portal area were detected by CD3 and CD8 staining. It was worth noting that the expression of TMEM199 was reduced in this index patient sample (Figure 1G), compared with the normal liver sample (donated by a surgical patient), which displayed a cytoplasmic positive pattern in hepatic and bile duct cells (Figure 1H).

### Molecular Genetic Analysis

**FIGURE 2:**
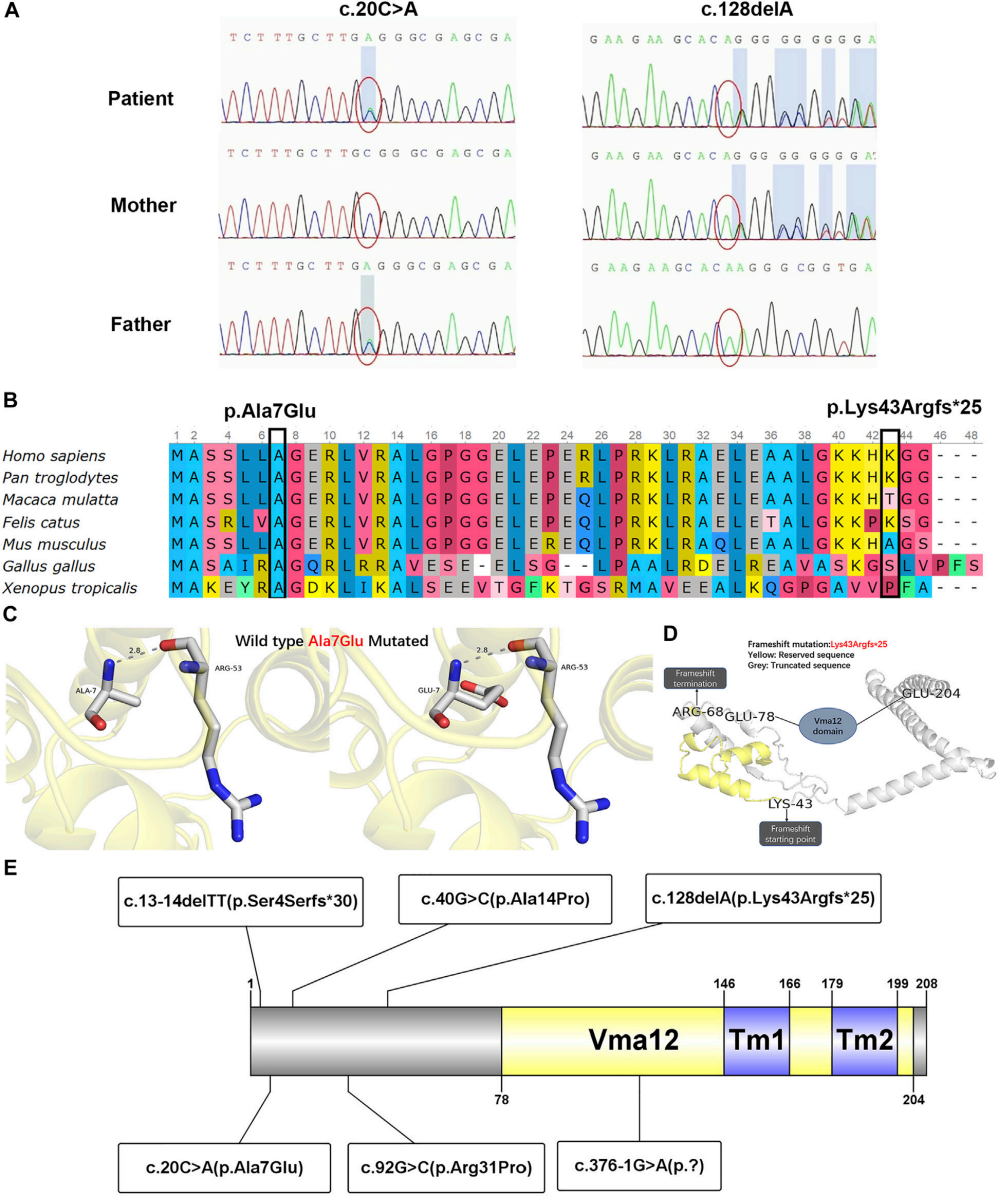
Genetic testing results, conservation of amino acid residues, protein modulation, and distribution of reported variants with the TMEM199 protein. **(A)** Sanger sequencing confirmation of c.20C > A and c.128delA mutation of *TMEM199* in the index case and his parents. **(B)** Conservation status of amino acid residues of the two variants across various species. **(C)** Wild and mutated types of the p.Ala7Glu variant compared by PyMol. **(D)** Spatial conformation of the frameshift mutation (p.Lys43Argfs*25) showed by protein modeling. **(E)** Illustration of TMEM199 protein domains, location of amino acid changes of the reported variants so far.

With a suspected diagnosis of Wilson disease according to the clinical manifestation, a multi-gene panel was screened. However, the absence of *ATP7B* mutation and normal DNA copy number variant (CNV) ruled out Wilson disease. Thereafter, two compound heterozygous variations, c.20C > A and c.128delA, were found in *TMEM199* gene exon 1 (Figure 2A) by WES. The c.20C > A was inherited from the healthy father, leading to a change of alanine to glutamate at amino acid position 7 (p.Ala7Glu), which was reported in the HGMD (Jansen et al., 2016). The c.128delA as a deletion mutation was inherited from the healthy mother, which caused a frameshift with an early termination (p.Lys43Argfs*25), which was not reported in the HGMD. The amino acid residue of the splice-site mutation (p.Ala7Glu) was conserved across various species, while the frameshift mutation (p.Lys43Argfs*25) was not conserved (Figure 2B). The CADD score of c.20C > A and c.128delA was 14.560 and 11.410 respectively, both predicted as a high impact by MSC. Both mutations of c.20C > A (functional test PS3 + absent from controls PM2 + homozygous variants and distribution in trans PM3_-_Strong + phenotype match PP4) and c.128delA (frameshift variant with LOF PVS1 + absent from controls PM2 + phenotype match PP4) were evaluated to be pathogenic according to the guideline of the ACMG. The nomenclature of variants was based on the recommendations of the Human Genome Variation Society (HGVS, http://www.hgvs.org/varnomen). Wild-type and mutated TMEM199 were modeled by PyMol (http://www.pymol.org) which showed no effect of Ala7Glu residue change on the polar contact with them around amino acid Arg53 (Figure 2C). Spatial conformation of the frameshift mutation (p.Lys43Argfs*25) predicted by protein modeling showed that the amino acid at position 43 moved backward 25 positions and then terminated, resulting in protein truncation, which contained a loss of the Vam12 domain (Figure 2D). Figure 2E illustrates the TMEM199 protein domains, location of amino acid changes of the reported variants so far.

## Discussion

CDGs are clinically and genetically heterogenous disorders sharing a primary defect of glycosylation. Mandato et al. (2006) detected aberrant glycosylation of transferrin (Tf) by electrospray ionization mass spectrometry (ESI-MS) in four children with cryptogenic chronic hypertransaminasemia and/or liver steatosis and fibrosis, leading to the discovery of a new type of CDG-X with unknown disease-causing genetic alterations. With the development of WES, researchers found two cases of CDG-X and one unrelated case with *TMEM199* heterozygous mutations (Vajro et al., 2018). In addition, Jansen et al. (2016) confirmed that the abnormal glycosylation of serum protein was caused by Golgi homeostasis defects, and mutations at different sites in the *TMEM199* gene were found in four patients. Two heterozygous mutations in *TMEM199* were detected in our patient with the abnormal liver function since birth. Based on the mutations of the *TMEM199* gene, all the eight patients were diagnosed with TMEM199-CDG. The main features of the eight patients are listed in Table 2.

**TABLE 2:** Main findings of the eight patients with TMEM199-CDG.

Patient	Patient 1	Patient 2	Patient 3	Patient 4	Patient 5	Patient 6	Patient 7	Patient 8
	Siblings				Siblings			
Reference	Jansen et al. (2016)	Jansen et al. (2016)	Jansen et al. (2016)	Jansen et al. (2016)	Vajro et al. (2018)	Vajro et al. (2018)	Vajro et al. (2018)	Current report
Gender	Male	Male	Male	Female	Female	Male	Male	Male
Age (years)	26	18	41	23	27	24	2	4
Country of origin	Greece	Greece	ND	Greece	Italy	Italy	Italy	China
Developmental delay	None	None	ND	Psychomotor disability	None	None	Mild delay of speech	Mild delay of psychomotor
Other manifestation	None	None	None	Hypotonia	None	None	None	Strabismus
Alanine aminotransferase (0–40 IU/L)	54	210	46–190	36–172	23–329	50–221	104–437	58.8–297.7
Aspartate aminotransferase (0–40 IU/L)	73	246	40–153	31–119	53–349	98–299	156–656	62.9–746.0
Alkaline phosphatase (42–383 IU/L)	745	1,162	365–718	132–1,528	1,140–1995	903–3990	713–1,235	410.0–1296.0
Total cholesterol (3.1–5.2 mmol/L)	6.5	8.7	5.3–8.7	6.0–6.6	7.8–8.8	5.7–6.2	3.6–4.1	6.09–9.30
LDL cholesterol (1.30–3.90 mmol/L)	4.86	7.16	4.70–5.84	4.16–4.42	6.20–6.62	4.55–4.58	2.48–2.53	4.36–7.17
HDL cholesterol (0.91–2.05 mmol/L)	ND	ND	ND	ND	1.27–1.40	1.16–1.19	1.27–1.40	1.01–3.03
Ceruloplasmin (0.22–0.58 g/L)	0.11	0.16	0.09	0.17–0.24	0.06–0.08	0.04–0.06	0.08–0.084	0.03–0.12
Serum copper (10.99–21.98 μmol/L)	ND	ND	ND	ND	<6.25	<6.25	<6.25	2.8–8.6
Coagulation parameters	Slightly decreased ATIII, FXI, FXIII, and protein S	Slightly decreased ATIII, FXI, FXIII, and protein S	ND	ND	Normal/borderline	Normal/borderline	Low ATIII activity	Prolonged APTT, increased D-dimer and decreased fibrinogen
Liver histology	Steatosis	Steatosis	Steatosis and minimal fibrosis	ND	Mild periportal fibrosis; focal steatosis	Mild periportal fibrosis; focal steatosis	ND	Steatosis and cirrhosis
Hepatic ultrastructure	ND	ND	Dilation and vesiculation of the Golgi and/or ER	ND	ND	ND	ND	ND
Immunohistochemistry of TMEM199	ND	ND	ND	ND	ND	ND	ND	Weak positive
Western blot of TMEM199	Reduced	Reduced	Reduced	ND	Undetectable	Undetectable	ND	ND
Zygosity	Homozygous	Homozygous	Compound heterozygous	Homozygous	Compound heterozygous	Compound heterozygous	Compound heterozygous	Compound heterozygous
Mutation sites	c.20C > A	c.20C > A	c.40G > C/c.376-1G > A	c.92G > C	c.13-14delTT/c.92G > C	c.13-14delTT/c.92G > C	c.13-14delTT/c.92G > C	c.20C > A/c.128delA
Amino acid change	p.Ala7Glu	p.Ala7Glu	p.Ala14Pro/ND	p.Arg31Pro	p.Ser4Serfs*30/p.Arg31Pro	p.Ser4Serfs*30/p.Arg31Pro	p.Ser4Serfs*30/p.Arg31Pro	p.Ala7Glu/p.Lys43Argfs*25
Glycosylation studies	Abnormal N- and O-glycosylation	Abnormal N- and O-glycosylation	Abnormal N-glycosylation	Abnormal N- and O-glycosylation	Type Ⅱ CDG pattern	Type Ⅱ CDG pattern	Type 2 CDG pattern	ND
Treatment	ND	ND	ND	Carbamazepine × 2 years at age 6 for attention deficit	Vitamin D × 1 year at age 4; penicillamine for 6 months at age 5 with no effects	None	None	Bicyclic alcohol, zinc sulfate and penicillamine for 1 year at age 4 with no effects

ND, not described.

The eight patients with TMEM199-CDG included seven Europeans (originating from Greece and Italy) and one Chinese. There were six male and two female patients, with an age range of 2–41 years, four of whom were two pairs of siblings. Seven patients had the disease onset since early childhood. Intellectual disability or mild language retardation was found in three patients, among whom one also presented with hypotonia, while one Chinese patient also presented with strabismus. All patients reported to date presented high fluctuating levels of alanine aminotransferase (23–437 IU/L, reference range 0–40 IU/L), aspartate aminotransferase (31–746 IU/L, reference range 0–40 IU/L), and alkaline phosphatase (132–3990 IU/L, reference range 42–383IU/L). Total cholesterol (5.3–9.3 mmol/L, reference range 3.1–5.2 mmol/L) and low-density lipoprotein cholesterol (1.16–7.17 mmol/L, reference range 1.30–3.90 mmol/L) were elevated in seven patients, while high-density lipoprotein cholesterol (1.01–3.03 mmol/L, reference range 0.91–2.05 mmol/L) was normal or slightly increased in four patients. Low levels of serum ceruloplasmin (0.03–0.24 g/L, reference range 0.22–0.58 g/L) and serum copper (2.80–8.60 umol/L, reference range 10.99–21.98 μmol/L) were found. The coagulation function was normal, close to borderline or slightly weakened in six patients. These findings indicate that TMEM199-CDG mainly affects the liver, and nervous and muscular systems may be involved as well.

Liver biopsies were performed in six patients, four of whom showed various degrees of steatosis and periportal fibrosis, and two only showed steatosis. Among them, one patient underwent the assessment of the ultrastructural characteristics of hepatocytes using a transmission electron microscope. Diffuse and severe vacuolization were observed to most likely derive from the rough ER, smooth ER, and/or Golgi apparatus (Jansen et al., 2016). Unlike the mild or minimal fibrosis manifested in European patients, the liver biopsy of the Chinese patient presented cirrhosis with bridging-like fibrosis and pseudo-lobule formation, which was assessed as stage 4 according to the Scheurer histopathologic scoring system (Scheuer, 1991). The presentation of cirrhosis in early childhood indicates that the severity of liver impairment caused by TMEM199-CDG may require more attention and early intervention.

WES demonstrated heterozygous *TMEM199* mutations in five patients and homozygous *TMEM199* mutations in three patients, including c.13-14delTT (p.Ser4Serfs*30), c.20C > A (p.Ala7Glu), c.40G > C (p.Ala14Pro), c.92G > C (p.Arg31Pro), c.128delA (p.Lys43Argfs*25), and c.376-1G > A (p.?) (Figure 2E). *TMEM199* (NM_152464.1, ENST00000292114) is located on chromosome 17 (chr17:26,684,604–26,690,705), containing six exons encoding a protein of 208 amino acids. The encoded TMEM199 (previously known as C17orf32), a transmembrane protein, is mainly located in the endoplasmic reticulum–Golgi apparatus intermediate compartment (ERGIC) and is involved in Golgi homeostasis. Moreover, as a human homolog of yeast Vma12p, TMEM199 participates in the formation of V-ATPase, which is the proton pump in the process of vesicle acidification via the secretory pathway (Miles et al., 2017). The homeostasis of Golgi apparatus cannot be maintained in TMEM199 deficiency due to the pH imbalance, leading to the abnormal glycosylation of serum proteins, which is closely related to the clinical manifestations of liver dysfunction in patients with TMEM199-CDG. In previously reported patients, glycosylation and functional studies showed aberrant N- and O-glycosylation as a type Ⅱ CDG pattern, and deficient incorporation of galactose and sialic acid as seen in other Golgi homeostasis defects, which was confirmed by metabolic labeling and restored by lentiviral transduction with wild-type TMEM199 (Jansen et al., 2016; Vajro et al., 2018). Hence, the hepatic phenotype with abnormal glycosylation is considered to be resulted from TMEM199 deficiency. Glycosylation defects and the abnormal protein function of the Chinese patient need to be determined in future studies. Additionally, the expression level of TMEM199 in the Chinese patient’s liver specimen detected by immunohistochemical staining was lower than normal control, which was consistent with the reduced or unmeasurable level of TMEM199 protein in skin fibroblasts detected using the Western blot in the European patients (Jansen et al., 2016; Vajro et al., 2018). The TMEM199 antibody (ab121907) is designed with a recombinant fragment corresponding to human TMEM199 amino acids at positions 7–143. Therefore, the unique c.128delA (p.Lys43Argfs*25) in *TMEM199* exon 1 in our patient caused a frameshift with an early termination, which was mainly responsible for the weak immunohistochemical signal.

Treatment and prognosis were reported in five patients, two of whom did not receive any medication. Despite the 2 years of carbamazepine usage for attention deficit starting at age 6, no other drugs were given to a Greek patient (Jansen et al., 2016). Vitamin D usage for 1 year starting at age 4 and penicillamine usage for 6 months starting at age 5 were reported in an Italian patient (Vajro et al., 2018), while the Chinese patient in the present study was given bicyclic alcohol, zinc sulfate, and penicillamine for 1 year initiating at age 4. However, it seemed that medical treatments had no definite effect on improving the liver function of these patients. Nevertheless, the previous patients were reported to have no deterioration over several decades, and the Chinese patient presented no significant disease progression during 1-year follow-up. Therefore, there was a view that TMEM199-CDG had a more favorable course to avoid worries of the patients and their parents and unnecessary repeated blood sampling (Vajro et al., 2018). As far as our case is concerned, the early emergence of cirrhosis makes the outcome of the disease not too optimistic; more cohort observations are needed to verify our findings.

## Conclusion

This report expands the phenotypic and genotypic spectra of TMEM199-CDG by presenting the first Chinese patient carrying strabismus, severe fibrosis, reduced expression of TMEM199, and a novel truncating mutation. Like the previously reported patients, this Chinese boy was monitored, without significant disease progression. But the appearance of cirrhosis in early childhood needs to be paid attention to, and more cases will help better understand the unclear course of this rare condition.

## Data Availability

The data presented in this study is included in the article/Supplementary Material. The DNA datasets are not readily available due to privacy restrictions, further inquiries should be directed to the corresponding authors.
